# A Standardized Extract of *Zingiber officinale* Roscoe Regulates Clinical and Biological Outcomes in Two Different EAE Mouse Models

**DOI:** 10.3390/biomedicines13020278

**Published:** 2025-01-23

**Authors:** Vittoria Borgonetti, Paolo Governa, Martina Morozzi, Chiara Sasia, Giacomina Videtta, Marco Biagi, Nicoletta Galeotti

**Affiliations:** 1 Department of Neuroscience, Psychology, Drug Research, and Child Health (NEUROFARBA), Section of Pharmacology, University of Florence, Viale G. Pieraccini 6, 50139 Florence, Italy; vittoria.borgonetti@unifi.it (V.B.); martina.morozzi@unifi.it (M.M.); chiara.sasia@unifi.it (C.S.); giacomina.videtta@unifi.it (G.V.); 2 Department of Biotechnology, Chemistry and Pharmacy, University of Siena, Via A. Moro 2, 53100 Siena, Italy; paolo.governa@unisi.it; 3 Department of Food and Drug, University of Parma, Parco Area delle Scienze 27/A, 43124 Parma, Italy; marco.biagi@unipr.it

**Keywords:** *Zingiber officinale* Roscoe, ginger extract, multiple sclerosis, EAE model, pain, disability, cytokines

## Abstract

**Background/Objectives**: Multiple sclerosis (MS) is a chronic inflammatory disease of the central nervous system characterized by demyelination and neuronal damage. Current MS therapies are unsatisfactory, and new therapies are encouraged. A correlation between nutritional intake and MS has been speculated. Supplementation of approved immunomodulatory therapy with herbal medicines possessing antioxidant and anti-inflammatory activities could provide benefits to MS patients. Ginger is one of the most widely consumed dietary supplements in the world, commonly used in traditional medicine. Studies demonstrated that ginger may also be beneficial in the management of neurodegenerative diseases. The aim of this study is to investigate the MS therapeutic potential of ginger. **Methods**: A standardized *Zingiber officinale* Roscoe extract (ZOE) was orally administered for 14 days. Two experimental autoimmune encephalomyelitis (EAE) models in mice were used. The PLP_139-151_-EAE relapsing-remitting model and MOG_35–55_-EAE chronic model. Clinical score, von Frey, hot plate, and rotarod tests were used for behavioral tests. ELISA and Western blotting were used to measure cytokines levels. Evans Blue content was determined spectrophotometrically. **Results**: ZOE attenuated motor disability and pain hypersensitivity in both models had no effect on body weight loss. ZOE reduced the blood–brain barrier (BBB) permeability in the PLP-EAE models and reduced levels of circulating cytokines (Il-6, IL-17) in the MOG-EAE model. ZOE attenuated spinal cytokines overexpression in both models. **Conclusions**: ZOE improves EAE symptoms and attenuates the proinflammatory response in both models, representing a promising nutraceutical support to the conventional therapeutic approach to MS.

## 1. Introduction

Multiple sclerosis (MS) is defined as an autoimmune disease characterized by strong inflammation, leading to myelin damage and neuron death, hence generating a dysfunction of the central nervous system [1]. In total, 85% of people with MS are initially diagnosed with relapsing-remitting MS, and can benefit from the use of immunomodulatory drugs that control the acute stages of the disease and delay the advent of secondary progressive MS [2]. Differently, chronic progressive MS patients are characterized by a progressive loss of neurologic function and an increase in disability over time. Research studies have speculated the existence of a relationship between nutritional intake and multiple sclerosis (MS) [3]. Lipid supplementation, as a source of substrates crucial in the remyelination process, including substrates for nervonic acid synthesis, might help achieve a more effective influence on CNS regeneration and the remyelination process [4,5]. Furthermore, supplementing the approved immunomodulatory therapy with herbal medicines possessing well-known antioxidant and anti-inflammatory activities could provide benefits to the quality of life of MS patients [6,7,8,9]. Encouraging results have been obtained with curcumin [10,11,12] and cannabis-based medicines [13,14,15]. However, the low bioavailability of curcumin [16,17] and the psychotropic effects of Δ^9^-tetrahydrocannabinol (THC) [18], limit their use in long-term clinical practice. Ginger (*Zingiber officinale* Roscoe) is one of the most widely consumed dietary supplements in the world, and it has been commonly used in traditional medicine for treating gastrointestinal disorders, as well as other ailments, such as colds, migraines, arthritis, and hypertension. In addition, accumulating studies have demonstrated that ginger may be beneficial for preventing and managing neurodegenerative diseases [19,20]. The FDA added ginger to the list of “generally recognized as safe” substances, up to a dose of 4 g/day [21]. Several preclinical [22,23] and clinical [24] studies revealed the ability of ginger supplementation to reduce inflammation. Interestingly, a clinical trial showed that the administration of 500 mg of ginger, three times daily for twelve weeks, ameliorated the Expanded Disability Status Scale and the Multiple Sclerosis Impact Scale in relapsing-remitting MS patients [25].

One of the main symptoms associated with MS is neuropathic pain. This is a hard-to-treat chronic pain condition that dramatically disrupts the patient’s normal daily activities. Previously, we showed the efficacy of a standardized pharmaceutical grade *Zingiber officinale* Roscoe extract (ZOE) in reducing allodynia and hyperalgesia in a mice model of neuropathic pain induced by nerve injury [26]. One of the main problems associated with the use of herbal medicine products is the variability in chemical composition. The composition of plant active constituents greatly varies with environmental factors (i.e., season, rain, irrigation, and temperature), chemical contamination (i.e., fertilizers, pesticides, heavy metals, etc.), and other soil problems, genes and health of the plant, the part of the plant used [27]. For this reason, using standardized pharmaceutical grade extracts that guarantee the consistency of their composition is pivotal for facilitating preclinical to clinical transition. The purpose of this work was to test the effect of a standardized ZOE in the experimental autoimmune encephalomyelitis (EAE) MS model in mice, induced by two different peptides: PLP_139–151_, mimicking the relapsing-remitting MS, and MOG_35–55_, reproducing symptoms associated with chronic progressive MS. The effect of ZOE on the EAE-associated motor and nociceptive phenotypes has been investigated, and the levels of circulating and spinal inflammatory cytokines were measured. The disruption of the blood–brain barrier (BBB) is an important pathogenetic feature of MS. BBB breakdown allows circulating leukocytes to penetrate the brain and leads to the generation of several anti-CNS autoantibodies in humans, playing a crucial role in the onset of the immune attack. Additionally, BBB leakage also contributes at later stages of the disease as a mechanism that sustains demyelination and neurodegeneration [28,29]. Thus, BBB integrity following ZOE treatment was investigated.

## 2. Materials and Methods

### 2.1. Animals and Ethics Approval

Mice for PLP_139–151_ (SJL females mice N = 41; 17–20 g, 10-week-old) and MOG_35–55_ (CB57BL/6 female mice N = 49; 17–20 g, 10-week-old) experiments were obtained from Envigo (Varese, Italy). Mice were housed in a temperature-controlled (23 ± 1 °C) animal facility (Centro Stabulazione Animali da Laboratorio, Ce.S.A.L.) with 12 h light and dark cycles (experiments in the light phase). Mouse food (standard laboratory diet) and water were freely available. The animals were given 24 h to acclimatize in the experimental room prior to testing. Animal experimental protocols were approved by the Animal Care and Research Ethics Committee of the University of Florence, Italy, and the Italian Department of Health (336/2022-PR) in accordance with international laws and policies (Directive 2010/63/EU of the European Parliament and of the council of 22 September 2010 on the protection of animals used for scientific purposes; Guide for the Care and Use of Laboratory Animals, US National Research Council, 2011). Animal studies follow the ARRIVE guidelines for experiments involving animals [30]. Efforts were made to minimize the number of animals used and their suffering.

### 2.2. EAE Model

Animals were immunized, as previously reported [31]. Briefly, in the flanks and at the base of the tail, with a total of 200 µg of PLP_139–151_ and MOG_35–55_ peptides (200 µg/mice, synthesized by EspiKem Srl., University of Florence, Firenze, Italy), they were emulsified in a complete Freund adjuvant (CFA; Merck, Milan, Italy), supplemented with 4 mg/mL of Mycobacterium tuberculosis (strain H37Ra; Difco Laboratories, Detroit, MI, USA) and then, administered subcutaneously in the flanks at the base of the tail, per animal. The control mice received CFA without antigen. Immediately after and 48 h later than immunization, all mice received an intraperitoneal injection of a 500 ng/100 µL pertussis toxin (Merck, Darmstadt, Germany). General health and body weight were assessed prior to immunization and every three days in a blinded manner until the completion of the study. Locomotor coordination and the nociceptive threshold were analyzed before onset (0) and regularly during the course of the disease after 3-, 7-, 10-, 14-, 21-, 24-, and 28-day post-immunization. All tests were performed with a blind procedure.

### 2.3. Drug Administration

Standardized *Zingiber officinale* extract (ZOE), obtained by supercritical CO_2_ extraction, was kindly provided by INDENA S.p.A. (Milan, Italy; batch number 46349). ZOE contains 24.73% total gingerols (10.07% 6-gingerol), 3.03% total shogaols (1.68% 6-shogaol), and 30.10% terpenoid-enriched fraction (28.20% zingiberene). The HPLC-DAD and GC-MS chemical characterization of ZOE is detailed in our previous paper [26] and reported in the Supplementary Materials (Supplementary Tables S1 and S2).

Mice were randomly assigned to each group by an individual other than the operator. ZOE was dissolved in sodium carboxymethyl cellulose (CMC 1% in saline) and orally administered by gavage before testing at the dose of 200 a mg/kg. The dose resulted in an active dose, as previously published in the dose–response curve [26,32]. ZOE (200 mg/kg) was administered once daily for 14 days starting from day 14 post-immunization. VEH/EAE group received an equivalent volume of the vehicle (CMC 1% in saline) on the same days. Each experimental group was composed of 15 mice, except for ZOE/PLP-EAE (n = 11) and ZOE/MOG-EAE (n = 19).

### 2.4. Behavioral Testing

Behavioral tests were analyzed before onset (0) and regularly during the disease after 3-, 7-, 10-, 14-, 21-, 24-, and 28-day post-immunization. All testing was performed with a blind procedure.

#### 2.4.1. Mechanical Allodynia

Mechanical allodynia was measured by using von Frey filaments (Ugo Basile, Bologna, Italy) with ascending force (0.07, 0.16, 0.4, 0.6, 1, 1.4, and 2.0 g) as previously reported [33,34]. The nociceptive response for mechanical sensitivity was expressed as a mechanical threshold in grams.

#### 2.4.2. Hot Plate Test

The thermal nociceptive threshold was measured using the Hot Plate Test (Ugo Basile, Bologna, Italy), as previously reported [34]. The latency time (expressed in seconds) to the response of the mice to the thermal stimulus was measured. An arbitrary cut-off of 45 s was adopted to avoid paw damage to the animal.

#### 2.4.3. Clinical Disease Score

Motor disability was assessed by detecting the clinical disease score. Scoring for EAE and sham mice was performed daily by means of a 5-point scale, as previously described [35]. EAE clinical disease was considered present with clinical scores ≥ 1, while scores ≤ 0.5 were classified as disease remission or absence.

#### 2.4.4. Rotarod Test

The rotarod test was used to evaluate the possible locomotor alterations induced by the EAE model. The integrity of motor coordination was assessed by counting the number of falls from the rod in 30 s, as described in [36]. The performance time was measured before and regularly after immunization.

#### 2.4.5. Weight Control

Another important point is the periodic control of weight, using a commercial scale. Weight was checked as a criterion to evaluate the welfare of animals using a commercial scale on each behavioral test day.

#### 2.4.6. Calculation of Index of Disease Progression for Each Behavioral Parameter

To understand the pathology trend for individual animals, we evaluated, for each animal, the index of disease progression (IDP) by calculating the ratio between the initial baseline value and the final recorded value registered in the PLP and MOG EAE models during the following behavioral test: Von Frey Test, Hot Plate Test, Rotarod Test, and the control of weight.

### 2.5. Evaluation of BBB Disruption in Brain and Spinal Cord

Quantitatively measuring the Evans blue content was performed to assess the degree of BBB disruption [37]. On day 28 post-immunization, a 2% Evans blue solution (Merck, Darmstadt, Germany) at a dose of 5.0 mL/kg, mice was intraperitoneally injected into mice and allowed to circulate for 4 h. Following this, mice were anesthetized (with Zoletil and xylazine) and transcardially perfused with saline to clear the Evans blue dye from the vascular system. The brain and lumbar spinal cord were then promptly harvested, and images were captured. Other peripheral tissues (liver, kidney, and spleen) from each animal (N = 3–5, for each experimental group) were extracted and used as a positive control for the correct injection of the probe. Tissues were homogenized in 2.5 mL of PBS and then mixed with an equal volume (2.5 mL) of 50% trichloroacetic acid to precipitate proteins (overnight, 4 °C). The samples were centrifuged at 10,000× *g* for 30 min, and the supernatants were collected. Absorbance at 610 nm was measured using a multiplate spectrophotometer. The Evans blue content was reported as micrograms per gram of brain and lumbar spinal cord, then normalized on the immunized-control group (VEH/EAE).

### 2.6. Cell Culture

BV2 murine immortalized microglial cells (mouse, C57BL/6, brain, microglial cells, Tema Ricerca, Genova, Italy; 16–20 passages) were thawed and kept in culture in a 75 cm^2^ flask (Sarstedt, Milan, Italy) in a medium containing RPMI, as previously described [26]. Human SH-SY5Y neuroblastoma cell line (A.T.C.C., Manassas, VA, USA; 10–20 passages) were cultured in a 1:1 combination of DMEM/F12 Ham’s nutrients medium (Merck, Darmstadt, Germany), under the same conditions used for BV-2 cells. Both cell lines were cultured at 37° C and 5% CO_2_ with daily change in culture medium, until confluence (70–80%). EDTA-trypsin solution (Sigma-Aldrich, Milan, Italy) was used to detach cells, and cell counting was performed using Trypan blue staining.

To induce neuroinflammation, BV2 cells were stimulated with LPS 250 ng/mL for 24 h. To evaluate the neuroprotective effect of ZOE and the main constituents, BV2 cells were pre-treated with ZOE, 6-gingerol (GIN), 6-shogaol (SHO), or the terpene-rich fraction (TER) at the respective concentrations in the extract, for 4 h and then stimulated with LPS 250 ng/mL for 24 h. The conditioned BV2 medium was collected and centrifuged (1000× *g* for 10 min, 37 °C) to isolate the supernatant. SH-SY5Y were sown in 96-well plates (2 × 10^4^ cell/well) and then treated with the BV2 conditioned medium for 24 h.

### 2.7. Cell Viability

The anti-neuroinflammatory and neuroprotective effect of ZOE and its constituents were evaluated by assessing the cell viability of LPS-stimulated BV2 cells and of the SH-SY5Y cells treated with the conditioned BV2 medium for 24 h. Unstimulated BV2 medium was used as control. The Cell Counting Kit-8 (CCK-8, Sigma-Aldrich, Milan, Italy), performed according to the manufacturer’s protocol, was used to assess cell viability. Absorbance at 450 nm was measured using a microplate reader. Each treatment was conducted in six replicates across three independent experiments.

### 2.8. Determination of TNF-α, IL-1β, IL-6, and IL-17 from Plasma

Blood samples (N = 3–7 per experimental group) were collected from anesthetized mice (via intraperitoneal injection of a Zoletil and xylazine cocktail) using the standard cardiac puncture technique. The samples were centrifuged at 3000× *g* for 10 min at 4 °C, and the plasma was subsequently collected. Protein levels of TNF-α, IL-1β, IL-6, and IL-17 were quantified using a non-competitive sandwich ELISA (Biolegend e-Bioscience DX Diagnostic, Monza, Italy), following the manufacturer’s instructions. Absorbance was measured at 450 nm using a microplate reader spectrophotometer.

### 2.9. Western Blotting Analysis

Spinal cords (n = 3–4 per experimental group) were immediately collected, homogenized in a lysis buffer, and centrifuged at 9000× *g* for 15 min at 4 °C. Protein quantification in the supernatant was assessed (protein assay kit, Bio-Rad Laboratories, Milan, Italy).

Protein samples (20 μg of protein/sample) were separated by 4–20% precast polyacrylamide gel electrophoresis (SDS-PAGE) and then blotted onto Midi Nitrocellulose membranes using Trans-Blot Turbo Transfer Starter System (Biorad Laboratories, Milan, Italy). Membranes were blocked with PBST containing 5% non-fat dry milk for 60 min, then incubated overnight at 4 °C with primary antibodies: IL-6 (1:500); IL-17 (1:500) (SantaCruz Biotechnology, Dallas, TX, USA). The next day, membranes were washed three times with PBST and incubated for 2 h with HRP-conjugated secondary antibodies, then detected by a chemiluminescence detection system (Life Technologies Italia, Monza, Italy). Signal intensity for each blot (pixels/mm^2^) was measured using ImageJ 2.14 (NIH). The signal intensity was normalized to that of total protein stained by Ponceau S. Results were achieved in three independent experiments (n = 3).

### 2.10. Statistical Analyses

The data and statistical analysis comply with the recommendations on experimental design and analysis in pharmacology [38]. For the in vivo test, two-way analysis of variance (ANOVA) followed by the Sidak’s multiple comparison test was used to compare locomotor behavior, pain behaviors, clinical score, and weight between groups during the time course of the experiment. Score value comparison between EAE- and sham mice was assessed using the non-parametric Wilcoxon test. For the IDP index for each parameter, we used the two-way ANOVA followed by uncorrected Fisher’s LSD comparison. For the ex vivo samples: For BBB permeability and cytokines dosages, we used the Unpaired t Test. The statistical significance criterion was *p* < 0.05. Outliers were identified and excluded from each experimental set using the ROUT method [39]. The results are expressed as mean ± SEM. The computer program GraphPad Prism version 10.1.1 (GraphPad Software Inc, San Diego, CA, USA) was used for the statistical analyses.

## 3. Results

### 3.1. Attenuation of EAE-Associated Symptom by Daily Administration of ZOE

**Figure 1:**
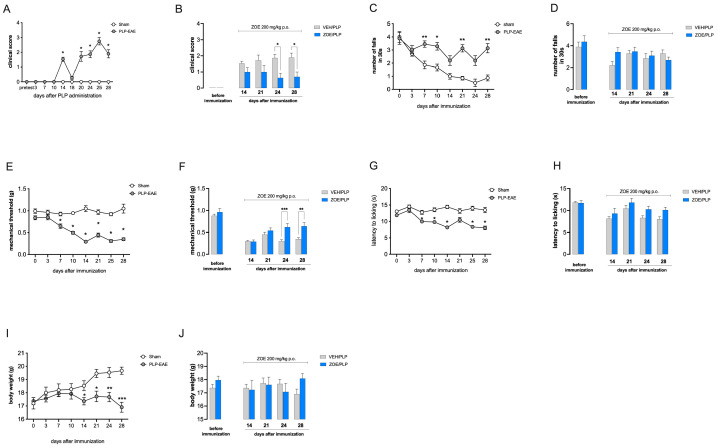
Oral ginger extract (ZOE) administration alleviates symptoms in PLP-EAE mice. (**A**) The clinical disease score of PLP_139–151_-EAE mice compared to the Sham mice showed a relapsing–remitting profile. * *p* < 0.05 vs. Sham. (**B**) Attenuation of motor disability by repeated ZOE treatment from day 24. * *p* < 0.05 versus VEH/PLP. (**C**) Locomotor impairment in PLP-EAE mice in the rotarod test in comparison with sham mice. * *p* < 0.05, ** *p* < 0.01 versus Sham. (**D**) ZOE showed a trend toward attenuation of rotarod performance impairment over time. (**E**) Time course evaluation of mechanical allodynia in EAE mice highlighting a relapsing–remitting nociceptive phenotype. * *p* < 0.05 vs. Sham. (**F**) ZOE ameliorated mechanical allodynia from day 24. ** *p* < 0.01, *** *p* < 0.001, versus Sham. (**G**) Time course evaluation of thermal hyperalgesia in PLP-EAE mice showing a relapsing-remitting nociceptive profile. * *p* < 0.05 versus Sham. (**H**) Repeated ZOE administration did not alleviate thermal hypersensitivity. (**I**) Measurement of body weight over time showing a weight loss of PLP-EAE mice in comparison with sham. * *p* < 0.05, ** *p* < 0.01, *** *p* < 0.001 versus Sham. (**J**) Lack of effects of ZOE treatment on body weight loss. ZOE treatment started on day 14 and ended on day 28. VEH: vehicle.

First, we tested ZOE in the relapsing-remitting EAE model (PLP-EAE). The key feature of this model is the fact that the animals did not show a progressive disease course, but had peaks of worsening (relapsing), alternating with the remitting phase of the disease (remitting). Fourteen days post-immunization (DPI), we started the administration of ZOE 200 mg/kg (ZOE/PLP). The PLP-EAE model showed motor disability with a relapsing-remitting phenotype, as shown by clinical score values (Figure 1A). ZOE repeated administration progressively attenuated motor symptoms with significant effects from 24 DPI (Figure 1B). PLP-EAE mice showed a relapsing-remitting locomotor alteration (Figure 1C). Even though not significant, ZOE showed a trend toward attenuation of this symptom (Figure 1D). The PLP-EAE model is also endowed with pain hypersensitivity, as demonstrated by the mechanical allodynia (Figure 1E) and thermal hyperalgesia (Figure 1G) that developed 14 DPI with a relapsing-remitting course. ZOE-treated animals significantly reduced mechanical allodynia at 24 and 28 DPI compared with vehicle-treated immunized animals (Figure 1F), whereas a non-significant trend toward attenuation was detected against thermal hyperalgesia (Figure 1H). PLP-EAE mice showed a progressive loss of body weight (Figure 1J). Overall, ZOE did not counteract the weight loss produced by the model (Figure 1J).

**Figure 2:**
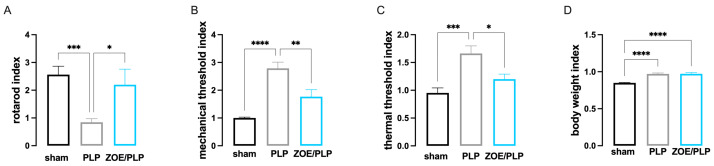
Evaluation of the Index of Disease Progression (IDP) in PLP-EAE mice. (**A**) IDP for locomotor impairment showed an attenuation of motor disability in ZOE-treated MOG-EAE mice. * *p* < 0.05, *** *p* < 0.001. (**B**) IDP for mechanical allodynia showed a reduction in the mechanical nociceptive phenotype after ZOE treatment. ** *p* < 0.01, **** *p* < 0.001. (**C**) IDP for thermal hyperalgesia highlighted a significant attenuation of thermal hypersensitivity by ZOE. * *p* < 0.05, *** *p* < 0.001. (**D**) IDP for body weight loss showed the lack of efficacy of ZOE administration. **** *p* < 0.0001. Sham and PLP groups receive vehicle oral treatment.

Calculation of the index of disease progression (IDP) by making a ration between the values recorded on day 0 and day 28 post-immunization registered per singular animals in each behavioral test performed, we confirmed that ZOE significantly reduced locomotor impairment (Figure 2A), mechanical allodynia (Figure 2B) and slightly decreased thermal hyperalgesia (Figure 2C). As previously said, no significant effects were observed for weight loss (Figure 2D) in the PLP-EAE model.

**Figure 3:**
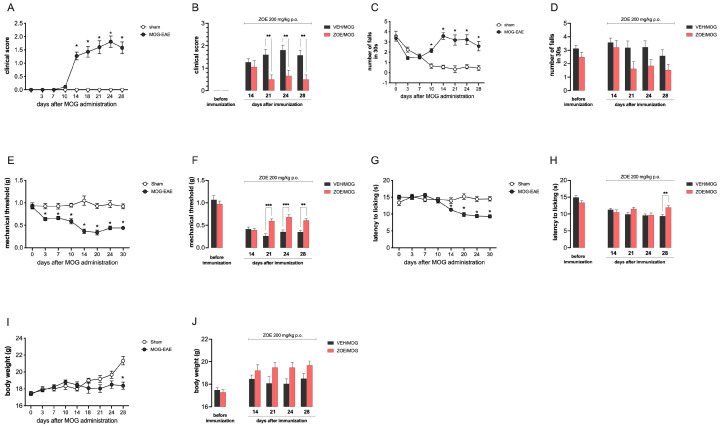
Attenuation by oral ginger extract (ZOE) of motor and pain symptoms in MOG-EAE mice. (**A**) The clinical disease score of MOG_35–55_-EAE mice compared to Sham mice showed a chronic progressive profile for motor disability. * *p* < 0.05 vs. Sham. (**B**) Reduction in clinical score values from day 21 by using repeated ZOE treatment. ** *p* < 0.01 versus VEH/PLP. (**C**) Progressive rotarod performance impairment in MOG-EAE mice in comparison with sham mice. * *p* < 0.05 versus Sham. (**D**) ZOE showed a non−statistically significant trend toward the attenuation of locomotor impairment over time. (**E**) Time course study of mechanical allodynia in MOG-EAE mice showed a chronic nociceptive phenotype. * *p* < 0.05 vs. Sham, (**F**) ZOE attenuated mechanical allodynia from day 21. ** *p* < 0.01, *** *p* < 0.001, versus Sham. (**G**) Time course evaluation of thermal hyperalgesia in MOG-EAE mice. * *p* < 0.05 vs. Sham. (**H**) Repeated ZOE administration attenuated thermal hypersensitivity on day 28. ** *p* < 0.01 (**I**) Body weight evaluation showed a weight loss in MOG-EAE mice in comparison with sham. * *p* < 0.05 versus Sham. (**J**) ZOE treatment effect on body weight loss. ZOE 200 oral administration started on day 14 and ended on day 28. VEH: vehicle.

Then, we tested ZOE 200 mg/kg in the chronic EAE model (MOG-EAE), which can represent a model for progressive multiple sclerosis, which is characterized by the progressive worsening of symptoms. MOG-EAE mice showed a progressive motor disability, as shown by the progressive worsening of clinical score values (Figure 3A) and rotarod locomotor impairment (Figure 3C). ZOE improved the general health condition compared to the VEH/MOG group, reducing clinical score values from 21 to 28 PDI (Figure 3B). No significant differences, but a slight effect was observed for the locomotor activity (Figure 3D) from day 21 to 28. This chronic model also developed mechanical allodynia (Figure 3D) and thermal hyperalgesia (Figure 3F) that were both attenuated by ZOE repeated treatment (Figure 3F,H), even if a prominent effect was detected toward mechanical allodynia (Figure 3F). ZOE 200 mg/kg administration did not seem to significantly control weight loss (Figure 3I,J), similarly to what observed in PLP-model.

**Figure 4:**
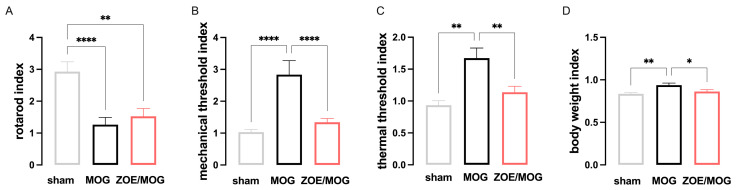
Evaluation of the Index of Disease Progression (IDP) in MOG-EAE mice. (**A**) IDP for locomotor impairment showed the lack of effect on controlling motor disability by ZOE treatment. ** *p* < 0.01, **** *p* < 0.0001. (**B**) IDP for mechanical allodynia indicated a reduction in the mechanical nociceptive phenotype in the ZOE-treated MOG-EAE group. **** *p* < 0.0001. (**C**) IDP for thermal hyperalgesia highlighted a significant attenuation of thermal hypersensitivity by ZOE. ** *p* < 0.01. (**D**) IDP for body weight loss showed the efficacy of ZOE administration on attenuating weight loss. * *p* < 0.05, ** *p* < 0.01. Sham and PLP mice receive vehicle oral treatment.

The IDP calculation confirmed the lack of significant effects in attenuating locomotor impairment (Figure 4A) and the attenuation of mechanical allodynia (Figure 4B) and thermal hyperalgesia (Figure 4C) by ZOE treatment. Conversely, IDP values highlighted that ZOE significantly attenuated weight loss (Figure 4D).

### 3.2. ZOE Reduced Damage to BBB Permeability in Spinal Cord PLP-EAE

**Figure 5:**
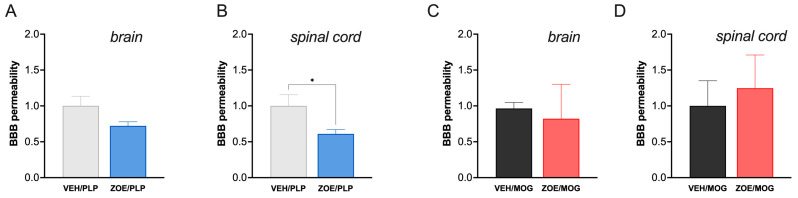
ZOE treatment effect on BBB permeability in the brain and spinal cord of EAE mice. Quantification of the amount of extravasated Evans Blue (EB) dye in the PLP-EAE brain (**A**) and the lumbar spinal cord (**B**) and in the MOG-EAE brain (**C**) and lumbar spinal cord (**D**) at 28 days after immunization. A significant attenuation of EB extravasation was obtained in the spinal cord of PLP-EAE mice treated with ZOE. * *p* < 0.05.

One of the major clinical symptoms of MS that is reproduced in EAE is the damage to the BBB. We quantified this damage using the Evans Blue (EB) dye penetration in the spinal cord and brain tissue. In these analyses, in the PLP-EAE model, ZOE significantly reduced EB content in spinal cord tissue (Figure 5A) compared to VEH/PLP and showed a no-significant tendency also in brain tissue (Figure 5B). Contrary to PLP, ZOE/MOG did not reduce BBB leaking compared to VEH/MOG group in the spinal cord (Figure 5C) or brain (Figure 5D) tissue in the MOG model.

### 3.3. Effect of ZOE on Plasma and Spinal Cytokines Levels in PLP/EAE and MOG/EAE Mice

**Figure 6:**
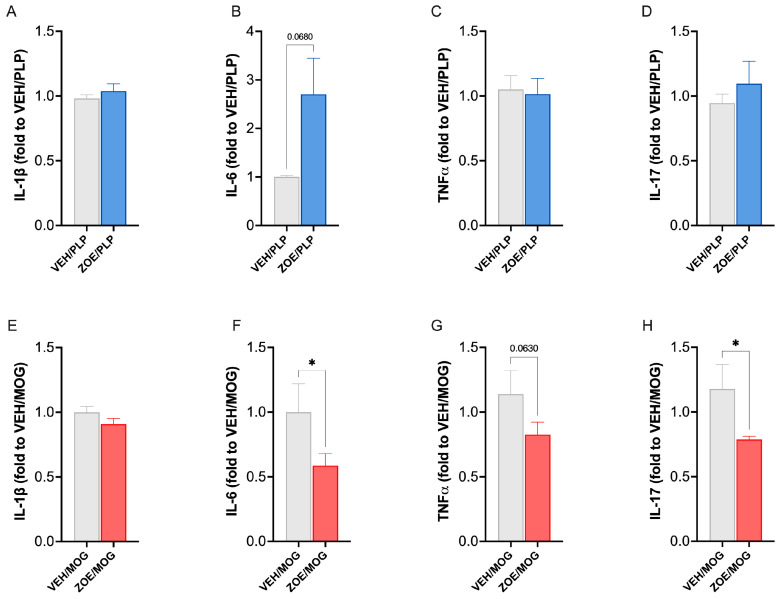
Effect of ZOE on plasma cytokines in PLP-EAE and MOG-EAAE mice. ZOE treatment in PLP-EAE mice did not significantly modify the levels of IL-1β (**A**), IL-6 (**B**), TNF-α (**C**), and IL-17 (**D**). In the MOG-EAE mice the plasma levels of IL-1β (**E**) and TNF-α (**G**), remained unmodified by ZOE administration while treatment reduced IL-6 (**F**) and IL-17 (**H**) levels. * *p* < 0.05.

In the EAE mouse model, plasma levels of main cytokines are increased [32]. Thus, we quantified the proinflammatory cytokines IL1β, IL6, TNF-⍺, and IL-17 in PLP-EAE and MOG-EAE plasma animals. No systemic anti-inflammatory effects of ZOE/PLP were observed compared to the VEH/PLP, showing no modification on plasma levels of IL-1ß (Figure 6A), TNF-⍺ (Figure 6C), or IL-17 (Figure 6D), but it appears to have an immunomodulatory action by showing a trend toward increasing levels of circulating IL-6 (Figure 6B). Consistently with the PLP model, no effect on IL1ß levels was observed (Figure 6E). However, differently from PLP, ZOE/MOG samples showed a reduced the plasma level of IL-6 (Figure 6F), and IL-17 (Figure 6H) compared to the VEH/MOG group and a trend toward attenuation of TNF-⍺ (Figure 6G).

**Figure 7:**
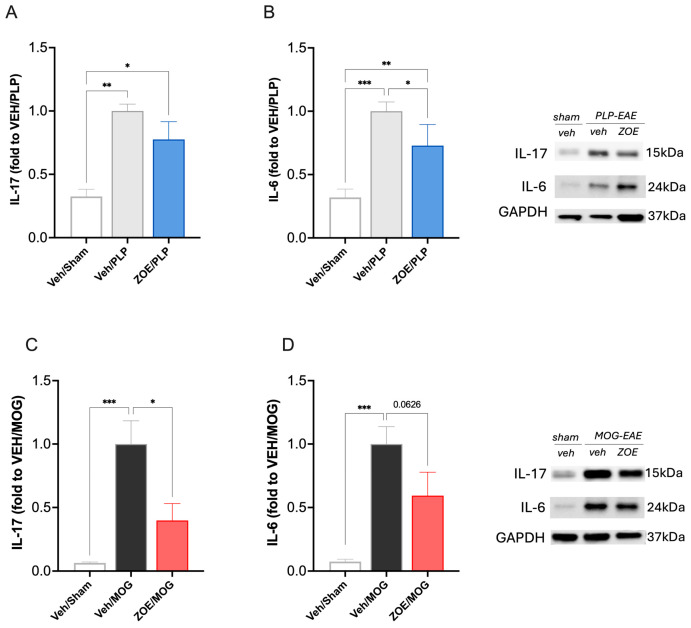
Effect of ZOE on spinal IL-17 and IL-6 expression in PLP-EAE and MOG-EAAE mice. Spinal levels of IL-17 and IL-6 ZOE were increased in the PLP-EAE mice (**A**,**B**) and in the MOG-EAE mice (**C**,**D**). ZOE treatment did not modify IL-17 (**A**) and reduced IL-6 (**B**) in PLP-EAE mice. In the MOG-EAE model, ZOE attenuated IL-17 (**C**) and IL-6 (**D**) protein expression. * *p* < 0.05, ** *p* < 0.01, *** *p* < 0.001.

Considering the efficacy of ZOE/MOG in reducing circulating proinflammatory cytokines IL-6 and IL-17, we evaluated its effect on spinal cytokine levels in both models. Protein quantification showed an increased expression of both cytokines in spinal samples from both models. Evaluation of IL-17 expression confirmed the lack of significant effect on PLP mice (Figure 7A) and the attenuation on MOG mice (Figure 7C). Concerning IL-6, a reduction in this cytokine was observed in PLP spinal samples (Figure 7B) and a trend toward attenuation of spinal overexpression in MOG samples (Figure 7D).

### 3.4. Contribution of ZOE Main Constituents to the Neuroprotective Effect

To assess the contribution of the main constituents of ZOE to its pharmacological activity in neuroinflammation-induced neurotoxicity, the effects of 6-gingerol (GIN), 6-shogaol (SHO), and the terpene-rich fraction (TER) at the concentration found in the active dose of ZOE were investigated.

**Figure 8:**
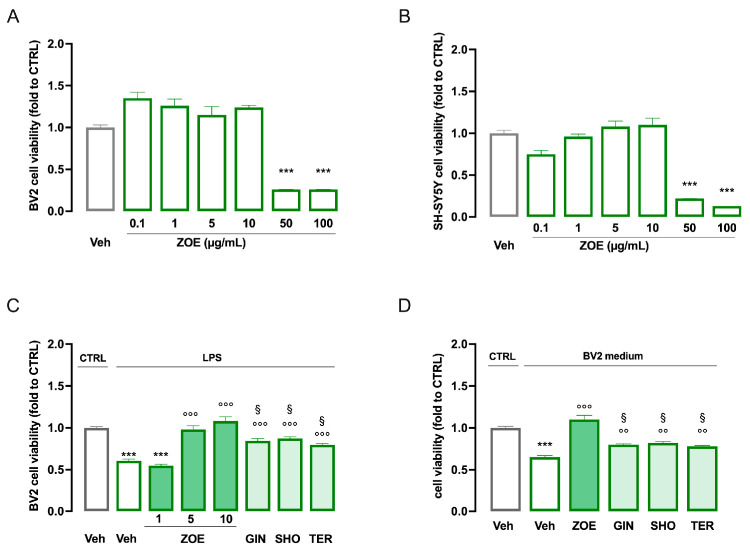
Neuroprotective effect of ZOE and main constituents. Dose–response curve for ZOE (0.1–100 µg/mL) on BV2 (**A**) and SH-SY5Y (**B**) cells. (**C**) Dose-dependent increase in cell viability by ZOE (1–10 μg/mL) on LPS-stimulated BV2 cells and partial effect of GIN (1 μg/mL), SHO (0.17 μg/mL) and TER (3 μg/mL). *** *p*< 0.001 vs. Veh; °°° *p* < 0.001 vs. LPS/Veh; § *p* < 0.05 vs. ZOE 10 µg/mL (**D**) neuroprotective effect of ZOE (10 µg/mL), GIN (1 μg/mL), SHO (0.17 μg/mL), and TER (3 μg/mL) on SH-SY5Y cells exposed to the conditioned medium from LPS-stimulated BV2. *** *p* < 0.001 vs. Veh; °°° *p* < 0.001, °° *p* < 0.01 vs. LPS/Veh; § *p* < 0.05 vs. ZOE 10 µg/mL.

First, dose–response curves for ZOE (0.1–100 µg/mL) were performed in microglia BV2 cells (Figure 8A) and neuronal SH-SY5Y cells (Figure 8B) to determine the maximal non-toxic concentration. Cell viability assessment showed that ZOE was not toxic up to 10 µg/mL. Higher doses drastically reduced cell viability and were not considered further in the study.

To evaluate the effect of ZOE on neuroinflammation, BV2 cells were stimulated with LPS (250 ng/mL) for 24 h. LPS treatment significantly reduced cell viability. Pretreatment with ZOE (0.1–10 µg/mL) led to a dose-dependent attenuation of the LPS effect, with the greatest effect observed at 10 µg/mL. To investigate the anti-neuroinflammatory effect of ZOE’s constituents, cells were pretreated with GIN (1 µg/mL), SHO (0.17 µg/mL), and TER (3 µg/mL). All constituents produced a partial protective effect of similar intensity (Figure 8C).

A reduction in cell viability was observed in SH-SY5Y cells after exposure to the LPS-stimulated BV2 medium, indicating the induction of neurotoxicity. Medium from BV2 cells pretreated with ZOE completely prevented cytotoxicity, while pretreatment with GIN, SHO, or TER partially protected the neuronal cells, with comparable efficacy (Figure 8D).

## 4. Discussion

Despite research progress and the development of increasingly effective therapies, a cure for MS has not been found, and MS patients continue to suffer from chronic progressive disability. In the efforts of identifying innovative and effective therapies, the present study examined the effect of a standardized pharmaceutical grade *Zingiber officinale* Roscoe extract (ZOE) on symptoms associated with MS in animal models.

MS is traditionally categorized into relapsing-remitting (RR) and primary progressive (PP) forms [40]. RRMS is the more prevalent phenotype (85–90% of patients), while PPMS accounts for 10–15% of cases and is characterized by a gradual accumulation of neurological disability, typically without relapses. The most widely used animal model for MS is the EAE model that reproduces phenotypes related to RRMS of PPMS [41]. Immunization of mice with the immunodominant epitope of PLP (PLP_139–151_) induces a relapsing-remitting disease course [42], while the immunodominant peptide MOG_35–55_ induces a chronic form of the disease [43].

The EAE model is widely used to experimentally replicate MS in laboratory animals. However, it has several limitations and notable differences when compared to human pathology. For example, EAE is induced artificially through immunization, and symptoms typically appear several weeks post-immunization. Spinal cord demyelination is a primary feature of the EAE model, whereas MS is a disease that predominantly affects the brain, with significant demyelination in the cerebral and cerebellar cortices. Moreover, the EAE model does not fully replicate key features of MS, such as the activation of the immune system [44]. Despite its limitations, the EAE animal model remains a crucial tool in the development of new therapeutic strategies for MS, as it is the model that most accurately mirrors the autoimmune pathogenesis of the disease.

The present study examined the effect of a standardized pharmaceutical grade *Zingiber officinale* Roscoe extract (ZOE) on symptoms associated with PLP_139–151_-induced EAE and the MOG_35–55_-induced EAE animal models. Both models are characterized by motor disability, body weight loss, central neuropathic pain, extensive inflammation in the white matter, and demyelination, whereas in the chronic MOG model, axonal degeneration was more prevalent [45]. Thus, investigating potential therapeutic interventions in both models can be helpful in highlighting potential differences in the efficacy of treatments toward different MS types. We found that repeated oral administration of ZOE 200 mg kg^−1^ reduced motor disability and pain phenotype in PLP/EAE and MOG/EAE models with no effect on body weight loss.

Motor disability is the main symptom occurring in EAE animals and MS patients. Experimentally, motor deficits have been defined by evaluating the clinical disease scoring, and ZOE reduced severity scores for motor disability in both models. These results are consistent with a previous study reporting the efficacy of a hydro-alcoholic extract of ginger in the MOG-EAE model [26,32,46]. These results have been implemented with rotarod measurements on EAE mice to add an objective test (unlike the EAE score) to the study of motor symptoms, and ZOE showed a lower efficacy in controlling locomotor impairment.

Pain is a severe and debilitating symptom experienced by MS patients, and it is one of the clinical signs of animals with EAE. Mechanical allodynia was the pain hypersensitivity symptom most prominently affected by ZOE treatment in both models. Indeed, ZOE reduced thermal hyperalgesia selectively in the MOG/EAE model. Consistently, in our previous work, ZOE reduced mechanical allodynia and thermal hyperalgesia in a mouse model of peripheral neuropathy [26,32]. The effect of the same ginger extracts on peripheral neuropathic pain was also observed in several preclinical studies [47,48,49]. However, to our knowledge, there are no known reports of examination of anti-allodynia or anti-hyperalgesia activity of ZOE in EAE models, and this study represents the first evidence of a pain-killing activity of a ginger extract.

MS is marked by a disrupted blood–brain barrier (BBB), infiltration of macrophages, T cells, and B cells into the central nervous system, along with local activation of microglia and astrocytes, all of which contribute to inflammation, demyelination, and neurodegeneration [50]. BBB breakdown is an early and key event in MS pathogenesis. In our study, we showed that ZOE reduced BBB damage in PLP-EAE mice but had no effect in the MOG-EAE model. However, the reduction in peripheral inflammatory cytokines represents a definite point of interest in reducing the progression of the disease, thereby helping prevent damage to the BBB and reducing neuroinflammation and damage to myelin. We then evaluated whether the anti-inflammatory properties of ginger might represent an important pharmacological mechanism in EAE models that might be involved in the attenuation of EAE-associated symptoms. Proinflammatory cytokines, such as IL-6, IL-1β, IL-17, and TNF-a, play an important role in both establishing and maintaining EAE and MS [50]. Quantification of plasma main cytokines showed the lack of significant effects of ZOE in PLP-EAE mice, while treatment reduced the levels of IL-6 and IL-17 in MOG-EAE mice. To further confirm the induction of an anti-inflammatory effect via the ZOE treatment, the spinal cord levels of cytokines were also analyzed to determine whether the changes observed in the peripheral blood were associated with the changes within the central nervous system. Data analysis revealed a general trend toward a reduction in cytokine levels, further supporting the hypothesis of an anti-neuroinflammatory effect of treatment.

Similarly to our results observed in MOG-EAE, the oral administration of a hydro-alcoholic extract of ginger (200–300 mg kg^−1^) reduced clinical scores compared to MOG-EAE control group mice, reducing inflammatory cytokines/chemokines (IL-17, IL-23, IL-33, CCL20, CCL22) and increasing anti-inflammatory factors (TGF-β, IL-12, IL-27) in serum and spinal cord tissues [46,51,52,53]. The different effects on some cytokine content between the literature study and our findings might be related to the slightly different chemical composition of the extracts obtained by different extraction methods, i.e., in the hydro-alcoholic extraction, the composition of the terpene component is quantitatively different from that in ours. Generally, studies conducted in other models of neurodegenerative diseases are mainly focused on ginger phenolic compounds, particularly 6-gingerol and 6-shogaol [19]. Han and collaborators [54], showed how 6-gingerol (i.p. 10 mg kg^−1^) significantly inhibited inflammatory cell infiltration from the periphery into the central nervous system and reduced neuroinflammation and demyelination in MOG mice. In another study, treatment of symptomatic EAE mice with 6-shogaol and 6-paradol (5 mg/kg/day, p.o.) significantly alleviated clinical signs of the disease, promoted remyelination, reduced cell infiltration in the spinal cord, and decreased levels of neuroinflammatory cytokines [55]. Studies previously conducted in our laboratory highlighted the importance of the terpene’s component in the pharmacological effect of ZOE in controlling neuropathic pain symptoms through a neuroprotective activity [26,32]. To evaluate the contribution of the main constituents of ZOE to its beneficial effects on neurological disturbances in EAE, we used cellular models of neuroinflammation and neurotoxicity. In LPS-stimulated microglia cells, we assessed the anti-neuroinflammatory effects of ZOE, while exposing SH-SY5Y cells to the BV2-conditioned medium, which revealed the neuroprotective effect of ZOE against neurotoxicity induced by proinflammatory microglia. Partial neuroprotective effects were observed with GIN, SHO, and TER, compared to the complete protection provided by ZOE, indicating that both phenolic and terpene fractions contribute to the beneficial activity of the extract.

Two recent clinical trials highlighted how ginger supplementation may be an effective adjuvant therapy for patients with relapsing-remitting MS (RR-MS). Foshati and collaborators [56] reported the supplementation of ginger (1500 mg/die for 12 weeks) was able to reduce gastrointestinal symptoms (nausea, frequency, and severity of constipation, bloating, and abdominal pain) in RR-MS patients. Moreover, in another study, the supplementation of ginger ameliorated some clinical signs in RRMS patients and reduced serum level of IL-17 [25]. The results from the present study show that ZOE improves EAE symptoms and reduces cytokines levels in both EAE models, also giving indication for efficacy in the progressive type of MS. Ginger is included in the FDA-approved list of “generally regarded as safe” [57]. This is a crucial aspect in the perspective of including ZOE as a supplement to the therapy of MS patients.

## 5. Conclusions

ZOE effectively improves MS symptoms and reduces neurological complications. Collectively, these findings suggest that ZOE, along with other classic and modern therapeutic interventions, represent a promising nutraceutical support to conventional MS therapeutic approach, particularly for its anti-allodynic effect.

## Data Availability

The original contributions presented in this study are included in the article/Supplementary Materials. Further inquiries can be directed to the corresponding author.

## Supplementary Materials

The following supporting information can be downloaded at: https://www.mdpi.com/article/10.3390/biomedicines13020278/s1; Table S1: HPLC-DAD quantification of 6-gingerol (6-GIN), 6-shogaol (6-SHO) and terpenoid-enriched fraction (ZTE) in the standardized extract from *Zingiber officinale* Roscoe rhizomes (ZOE).; Table S2: Chemical composition of the volatile compounds in the ethanolic ginger extract.

## Abbreviations

The following abbreviations are used in this manuscript:

MS	Multiple Sclerosis
EAE	Experimental Autoimmune Encephalomyelitis
ZOE	*Zingiber officinale* Roscoe extract
BBB	Blood–Brain Barrier
