# Supplementary material for: A Standardized Extract of Zingiber officinale Roscoe Regulates Clinical and Biological Outcomes in Two Different EAE Mouse Models
Source: Biomedicines. 2025 Jan 23;13(2):278. doi: 10.3390/biomedicines13020278 (PMC11852164; doi:10.3390/biomedicines13020278)
Supplement: Supplementary file 1 [file biomedicines-13-00278-s001.zip › biomedicines-3429059-supplementary.pdf]

Table S1
HPLC-DAD quantification of 6-gingerol (6-GIN), 6-shogaol (6-SHO) and terpenoid-enriched fraction (TER) in the standardized extract from Zingiber officinale Roscoe rhizomes (ZOE).

COMPOUND	%
6-GIN	10.07±0.51
6-SHO	1.68±0.33
TER	30.10±1.50

Data are expressed as %±standard deviation

Table S2. Chemical composition of the volatile compounds in the ethanolic ginger extract. Data are expressed as % area ± SD.

Peak number	compound	*LRI*	% Area
1	ethyl butanoate	805	1.7 ± 0.9
2	α-pinene	933	1.5 ± 0.2
3	camphene	947	4.1 ± 0.8
4	β-pinene	976	0.2[a]
5	β-myrcene	988	0.3[a]
6	octanal	992	0.8 ± 0.1
7	α-phellandrene	1005	0.7 ± 0.2
8	β-thujene	1029	4.6 ± 1.1
9	1,8-cineole	1031	2.3 ± 0.4
10	terpinolene	1088	0.3[a]
11	β-linalool	1101	0.9[a]
12	endoborneol	1166	1.4 ± 0.1
13	terpinen-4-ol	1178	0.3[a]
14	α-terpineol	1191	0.8 ± 0.3
15	verbenone	1208	0.7 ± 0.5
16	neral	1246	0.9[a]
17	geraniol	1260	0.4 ± 0.2
18	geranial	1275	1.5[a]
19	α-copaene	1380	0.6 ± 0.2
20	β-elemene	1386	0.6[a]
21	α-cedrene	1395	0.8 ± 0.3
22	α-gurjunene	1409	0.6 ± 0.3
23	α-bergamotene	1439	0.7 ± 0.2
24	β-farnesene	1461	0.8 ± 0.1
25	aromadendrene	1468	0.5 ± 0.2
26	germacrene D	1484	0.6 ± 0.2
27	α-curcumene	1488	6.2 ± 0.1
28	zingiberene	1502	28.2 ± 0.3
29	γ-muurolene	1505	2.3 ± 0.2
30	(E,E)-α-farnesene	1513	6.9[a]
31	β-bisabolene	1515	4.3[a]
32	β-sesquiphellandrene	1531	11.0 ± 0.2
33	δ-cadinene	1538	0.5[a]
34	(E)-nerolidol	1560	0.1[a]
35	germacrene B	1568	0.4 ± 0.1
	**TOTAL**		88.6 ± 0.8

[a] SD < 0.05
